# Chitosan Oligomer as a Raw Material for Obtaining Polyurethane Foams

**DOI:** 10.3390/polym15143084

**Published:** 2023-07-18

**Authors:** Anna Strzałka, Renata Lubczak, Jacek Lubczak

**Affiliations:** 1 Doctoral School of Engineering and Technical Sciences, Rzeszow University of Technology, Al. Powstancow Warszawy 6, 35-959 Rzeszow, Poland; amstrzalka1@interia.pl; 2 Faculty of Chemistry, Rzeszow University of Technology, Al. Powstancow Warszawy 6, 35-959 Rzeszow, Poland; rlubczak@prz.edu.pl

**Keywords:** chitosan oligomer, hydroxyalkylation, analysis of reaction, polyols, polyurethane foams

## Abstract

Decreasing oil extraction stimulates attempts to use biologically available sources to produce polyols, which are the basic components for obtaining polyurethane foams. Plants are inexhaustible source of oils, sugars, starches, and cellulose. Similar substrates to obtain polyols are chitosans. Commercially available modified chitosans are soluble in water, which gives them the possibility to react with hydroxyalkylating agents. We used a water-soluble chitosan previously to obtain polyols suitable for producing rigid polyurethane foams. Here, we described hydroxyalkylation of a low-molecular-weight chitosan (oligomeric chitosan) with glycidol and ethylene carbonate to obtain polyols. The polyols were isolated and studied in detail by IR, ^1^H-NMR, and MALDI–ToF methods. Their properties, such as density, viscosity, surface tension, and hydroxyl numbers, were determined. The progress of the hydroxyalkylation reaction of water-soluble chitosan and chitosan oligomer with glycidol was compared in order to characterize the reactivity and mechanism of the process. We found that the hydroxyalkylation of chitosan with glycidol in glycerol resulted in the formation of a multifunctional product suitable for further conversion to polyurethane foams with favorable properties. The straightforward hydroxyalkylation of chitosan with glycidol was accompanied by the oligomerization of glycidol. The hydroxyalkylation of chitosan with glycidol in the presence of ethylene carbonate was accompanied by minor hydroxyalkylation of chitosan with ethylene carbonate. The chosen polyols were used to obtain rigid polyurethane foams which were characterized by physical parameters such as apparent density, water uptake, dimension stability, heat conductance, compressive strength, and heat resistance at 150 and 175 °C. The properties of polyurethane foams obtained from chitosan-oligomer and water-soluble-chitosan sources were compared. Polyurethane foams obtained from polyols synthesized in the presence of glycerol had advantageous properties such as low thermal conductivity, enhanced thermal resistance, dimensional stability, low water uptake, and high compressive strength, growing remarkably upon thermal exposure.

## 1. Introduction

Polyols belong to the group of macromolecular compounds with at least two hydroxyl groups. They are important substances in the chemical industry, used to obtain polyurethanes [1]. Polyurethane foams (PUFs) have been present in the industry for at least 80 years and are polymers of wide application. They are used in construction, automotive, and furniture industry and daily used items in households [2]. These materials are obtained in foaming reaction between polyols and diisocyanates in the presence of water, catalyst, and various additional materials as surfactants and self-extinguishing and blowing agents [3]. At present, most polyols used to fabricate PUFs are obtained from petrochemical sources [2].

**Scheme 1:**
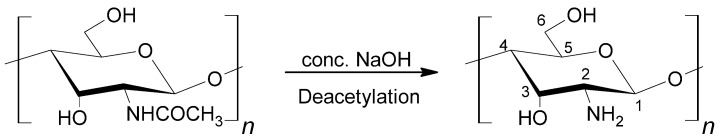
Deacetylation of chitin.

The dynamic economic growth of the modern world resulted in an increased demand for fossil fuels such as oil and gas, which are nonrenewable sources used in the chemical industry. The emergence of trends related to the so-called “green chemistry” and the awareness of the decreasing oil extraction prompted the search for the polyol substrates of biological sources available in the earth’s biosphere. The widespread use of chemical industry products raised the standard of living of wide circles of society. However, such a large development and progress in technology have also contributed to the chemical contamination of the environment. A high demand for foam products prompted chemists to search for the appropriate natural sources to produce polyols. The main goal is to limit fossil fuel extraction and conversion in order to diminish the natural environment degradation by introducing easily degradable polyols which are more susceptible to decomposition. Polyols based on natural renewable resources create new application possibilities not only because of their wide availability around the world but also because of their relatively low cost and potential biodegradability. Such substrates as lipids, including plant oils such as sunflower, palm, and soy oils [4,5], biomass components, such as lignin–cellulose and tannins [6], or polysaccharides such as starch and cellulose [7,8] are used to obtain polyols. Within these groups, chitosan (CS) is particularly interesting. CS can be obtained from wastes associated with shellfish in the food industry such as crabs and shrimp [9]. Additionally, CS is present in the cell walls of the shells of some fungi and insects [10]. CS can be obtained from chitin by the deacetylation reaction [9,11] (Scheme 1).

There are various kinds of CS available commercially, which differ in molecular mass and deacetylation degree. The low solubility of CS requires its various chemical modifications, which increase the scope of its application [12,13,14]. There are some functional groups such as the amine group at C-2 and primary and secondary hydroxyl groups at C-6 and C-3, respectively, which are convenient for the CS derivatization. CS is a linear polymer, composed of repeatable β-(1,4)-D-glucosamine and N-acetyl-D-glucosamine units remaining after a non-deacetylated chitin [11]. CS is a nontoxic, biodegradable, and biocompatible polymer. Therefore, CS has found applications in medicine, tissue engineering, pharmacy, and cosmetology [10,14,15,16,17,18]. Deacetylated CS can form polycationic species in acidic solutions, and thus, it has also found applications in water treatment [19,20,21]. Chitosan-derived materials find growing applications in medicine [22,23]. For instance, chitosan was used as a carrier for the *Indigofera intricata* plant extract with anticancer activity [24]. Chitosan nanocomposites have also been demonstrated as an effective material to remove hexavalent chromium from wastewater [25]. Carboxymethyl chitosans were evidenced as composite scaffolds for vasculogenesis [26]. Chitosan biopolymers were also used to monitor gamma radiation doses [27]. Chitosan-coated magnetite nanoparticles were shown to adsorb divalent copper ions [28].

Due to the presence of free hydroxyl groups in the CS, the hydroxyalkylation reaction is possible, which enables the introduction of additional polycarbonate chains with the hydroxyl groups. This was demonstrated in some CS derivatives obtained by a reaction with propylene oxide [29,30], which were then grafted on peptides (collagen and nisin), and the latter composites can find their applications in the healing bandages in medicine. Glycidol (GL) is also a convenient hydroxyalkylating agent for CS derivatization. It was demonstrated that the reaction of CS with GL and dodecyl alcohol led to the formation of a polymeric drug delivery system applied further to encapsulate quercetin and tested as an anticancer drug [31].

The hydroxyalkylation of CS makes it possible to obtain polyols suitable for the production of PUFs similar to the results demonstrated in the case of the hydroxyalkylation of cellulose and starch [7,8]. Chitosan may be used as a source to obtain polyols, alternative to petrochemical sources, provided that it can be liquefied, which can be achieved be hydroxyalkylation [32,33]. The application of chitosan in the fabrication of polyurethane foams creates new directions for the use and management of readily available chitosan waste accompanying the food industry. At the same time, it fills the research gap constituting almost a complete lack of information in the literature about the possibility of using chitosan in the synthesis of polyurethane foams. There are two reports on the synthesis of polyols from CS. Fernandes et al. [32] reported that a reaction of chitin and CS activated with KOH with the excess of ethylene oxide led to a polyol suitable as a semi-product to obtain polyurethanes and polyesters. Their protocol required a burdensome CS-swelling step with the use of NaOH in alcohol. Another protocol leading to CS-derived polyols was established in our laboratory [33] by the hydroxyalkylation of CS with GL and ethylene carbonate (EC). It should be mentioned that generally the difficulty of the CS hydroxyalkylation is due to its low solubility in organic solvents and in water. Therefore, a high-molecular-weight CS has not been hydroxyalkylated so far. In the work described in [33], we used the so-called water-soluble chitosan (CS_WS_), which was characterized by a better solubility, and we subjected it to the hydroxyalkylation with GL and then EC to obtain the expected polyols. This time, we focused on another kind of the CS, the chitosan oligomer (CS_O_). Detailed studies were performed to investigate the reaction of CS_O_ in the presence of glycerol (GLYC) and in the absence of GLYC, and to compare these reaction systems with a previously elaborated analogous reaction with a CS_WS_ as a substrate. We obtained polyols and then PUFs thereof based on an oligomeric CS. The properties of the obtained PUFs were compared to those of the PUFs previously obtained from a CS_WS_ [33].

Recently, we found that the presence of chitosan in the structure of PUFs renders more thermally resistant PUFs compared to the classic PUFs which lose their functional properties already at a temperature of 90–110 °C. Chitosan-based PUFs can withstand long-term heating even at 175 °C [33]. The degradation of polyurethane foams at elevated temperatures makes the experimental measurement of their thermophysical properties possible only at up to 150–200 °C. However, knowledge is needed about PUFs at higher temperatures to fully understand their behaviour and to develop advanced numerical models for the analysis and design of structures. In [34], a numerical inverse analysis procedure (based on the minimization of a discrete least squares function and a one-dimensional nonlinear finite element model of heat transfer together with experimental temperature distribution) to determine the effective values of the thermophysical properties of rigid polyurethane (PUR) foams was presented, which might be a specific route to analyze the chosen PUFs, especially those highly resistant to heating.

## 2. Materials and Methods

### 2.1. Materials

The following materials were used in the work: water soluble chitosan, degree of deacetylation DD = 86%, viscosity molecular weight *Mv* ~ 141,250 Da, (CS_WS_, Biosynth-Carbosynth, Staad, Switzerland); chitosan oligomer, degree of deacetylation DD = 68%, viscosity molecular weight *Mv* ~ 8700 Da, (CS_O_, Biosynth-Carbosynth, Staad, Switzerland); glycidol (GL, pure 98%, Sigma-Aldrich, Taufkirchen, Germany); ethylene carbonate (EC, pure ≥ 99%, Fluka, Buchs, Switzerland); potassium carbonate (anal. grade 100%, POCH, Gliwice, Poland); polymeric diphenylmethane 4,4′–diisocyanate (pMDI, Merck, Darmstadt, Germany); triethylamine (TEA, anal. grade ≥ 99%, Fluka, Buchs, Switzerland); surfactant Silicon L-6900 (pure, Momentive, Wilton, CT, USA); and glycerol (GLYC, anal. grade 99.5–100%, POCH, Gliwice, Poland).

### 2.2. Syntheses of Polyols

#### 2.2.1. Synthesis 1: Polyol (CS_O_ + GLYC + GL) + EC

A total of 8 g CS_O_, 80 g GL, and 60 g GLYC was placed in 250 cm^3^ three-necked round bottom flask equipped with reflux condenser, mechanical stirrer, and thermometer. The mixture was heated to 155 °C while stirring. At this point, an exothermic effect was observed, which lasted ca. 15 min. After that, the mixture was heat up to 190 °C and maintained until full consumption of GL. A total of 160 g EC and 1.0 g potassium carbonate was added to this semi-product (CS_O_ + GL + GLYC) as catalysts.

The mixture was reheated to 180–185 °C until EC reacted completely (ca. 14 h). The polyol (CS_O_ + GLYC + GL) + EC was obtained.

#### 2.2.2. Synthesis 2: Polyol (CS_O_ + GL) + EC

A total of 12 g CS_O_ and 45 g GL was placed in 250 cm^3^ three-necked round bottom flask equipped with reflux condenser, mechanical stirrer, and thermometer. The mixture was gently heated with vigorous stirring up to 190 °C and maintained at this temperature until complete consumption of GL. The semi-product (CS_O_ + GL) was cooled down to 80 °C, and 120 g EC and 1.5 g potassium carbonate as catalyst were added. The mixture was reheated to 180 °C and maintained as such to complete reaction of EC. After 14 h, the polyol (CS_O_ + GL) + EC was obtained.

#### 2.2.3. Synthesis 3: Polyol (CS_O_ + GL + EC)

A total of 20 g CS_O_, 75 g GL, and 200 g EC was placed in three-necked round bottom flask equipped with reflux condenser, mechanical stirrer, and thermometer. The mixture was heated to 140–145 °C while slow solubilization of CS was observed. Then, the reaction temperature was raised slowly to about 160 °C, where it is limited by the GL boiling point. The temperature was further slowly raised to 190 °C and maintained for half an hour in order to allow GL to react completely. Afterwards, the mixture was cooled down to 80 °C, 4 g of potassium carbonate was added, and the mixture was heated at 180 °C until EC reacted completely (ca. 6 h) to eventually obtain the (CS_o_ + GL + EC) polyol.

Based on mass balance, we concluded that the yield of reaction related to GL was always 100%. During the hydroxyalkylation with EC, the hydroxyalkylating agent decomposed partly at elevated temperature to ethylene oxide and carbon dioxide; thus, the yield of reaction for syntheses 1, 2, and 3 was 92.5, 93.4, and 96.2%, respectively.

### 2.3. Analytical Methods

Deacetylation degree of chitosans was determined according to the results of elemental analysis as it was described in [35]. Molecular mass was determined by viscosimetric method at 30 °C using Mark–Houwink equation as described in [36]:

[η] = k M_υ_^α^ (1)

where [η] is the intrinsic viscosity, and Mυ is the viscosity-average mass weight. Values k, α are constants that are characteristic for a particular polymer–solvent system at a specific temperature.

k = (1.64 × 10^−30^) × (DD^14^) [cm^3^/g] (2)

α = (−1.02 × 10^−2^) × (DD) + 1.82 (3)

where DD is the % degree of deacetylation.

The reaction of the CS and GL mixture was monitored by epoxide number determination using hydrochloric acid in dioxane [37]. Specifically, 25 cm^3^ of hydrochloric acid solution in dioxane (1.6 cm^3^ in 100 cm^3^ dioxane) was added to a 0.5 g mass sample. The excess of HCl was then titrated with 0.2 M NaOH in methanol in the presence of *o*-cresol red as an indicator. The progress of the hydroxyalkylation reaction with EC was monitored using barium hydroxide method described in [38]. In particular, the samples of 0.1–0.5 g mass were treated with 2.5 cm^3^ 0.15 M Ba(OH)_2_ and then titrated with 0.1 M HCl in the presence 0.2% thymolfhthalein in alcohol. Finally, the hydroxyl number (HN) of polyol was determined by acylation with acetate anhydride in dimethylformamide [39]. Thus, 1 g sample was heated with 20 cm^3^ acetylating mixture (acetic anhydride and dimethylformamide at 23:77 *v*:*v* ratio) for 1 h at 100 °C. The excess of anhydride was titrated with 1.5 M NaOH_aq_ in the presence of phenolphthalein. The ^1^H-NMR spectra of reagents were recorded using a 500 MHz Bruker UltraShield instrument in DMSO-d_6_ and D_2_O with hexamethyldisiloxane as internal standard. IR spectra were registered on ALPHA FT-IR BRUKER spectrometer in KBr pellets or by ATR technique. The samples were scanned 25 times in the range from 4000 to 450 cm^−1^ at 2 cm^−1^ resolution. MALDI–ToF (Matrix-associated laser desorption/ionization and time-of-flight spectrometry) spectra of polyols were obtained on Voyager-Elite Perceptive Biosystems (US) mass spectrometer working in a linear mode with delayed ion extraction, equipped with nitrogen laser working at 352 nm. The method of laser desorption from gold nanoparticles (AuNPET LDI MS) was applied [40]. Some observed peaks corresponded to the molecular K^+^ (from catalyst) ions. The samples were diluted with methanol to 0.5 mg/cm^3^.

### 2.4. Physical Properties of Polyol

Density, viscosity, and surface tension of polyols were determined with pycnometer, Höppler viscometer (typ BHZ, prod. Prüfgeratewerk, Germany), and by the detaching ring method, respectively.

### 2.5. Polyurethane Foams

Foaming of polyols was performed at 500 cm^3^ cups at room temperature. The foams were prepared from 10 g of polyol, to which 0.29–0.58 g of surfactant (Silicon L-6900) and 0.5–1.3 g of TEA as catalyst and water (2–4%) as blowing agent were added. After homogenization, the pMDI was added in the amount of 13.5–18.0 g. The commercial isocyanate containing 30% of tri-functional isocyanates was used. The mixture was vigorously stirred until creaming began. The materials were then seasoned at room temperature for 3 days. The samples for further studies were cut from the obtained foam.

### 2.6. Properties of Foams

The apparent density [41], water absorption [42], dimensional stability at 150 °C temperature [43], thermal conductivity coefficient (IZOMET 2104, Bratislava, Slovakia), and compressive strength [44] of PUFs were measured. The apparent density of PUFs was calculated as the ratio of PUF mass to the measured volume of PUF sample in cube of 50 mm edge length. Water volume absorption was measured on cubic samples of 30 mm edge length by full immersion of PUFs in water and mass measurement after 5 min, 3 h, and 24 h. Dimensional stability was tested on samples of 100 mm × 100 mm× 25 mm size. The thermal conductivity coefficient was measured at 20 °C after 72 h of PUF conditioning. The needle was inserted 8 cm deep into a cylindrical PUF sample 8 cm in diameter and 9 cm high. Compressive strength was determined using burden causing 10% compression of PUF height related to initial height (in accordance with the PUF growing direction). The thermal resistance of foams was determined both by static and dynamic methods. In the static method, the foams were heated to 150 and 175 °C with continuous measurement of mass loss and determination of mechanical properties before and after heat exposure. The 100 mm× 100 mm × 100 mm cubic samples were used to determine the static thermal resistance and compressive strength. In dynamic method, thermal analyses of foams were performed in ceramic crucible at 20–600 °C temperature range with about 100 mg of sample under air atmosphere, using a Thermobalance TGA/DSC 1 derivatograph, Mettler, at a 10 °C/min heating rate. Topological pictures of PUFs were recorded for cross-sections of PUF samples. The pictures were analyzed using a Panthera microscope (prod. Motic, Wetzlar, Germany) with 4 × 0.13, 10 × 0.30 lenses and developed with the help of the Motic Multi-Focus Professional 1.0 software enabling the merging and manipulation of images with adjustable lensing planes.

## 3. Results and Discussion

### 3.1. Obtaining of Polyols

**Scheme 2:**
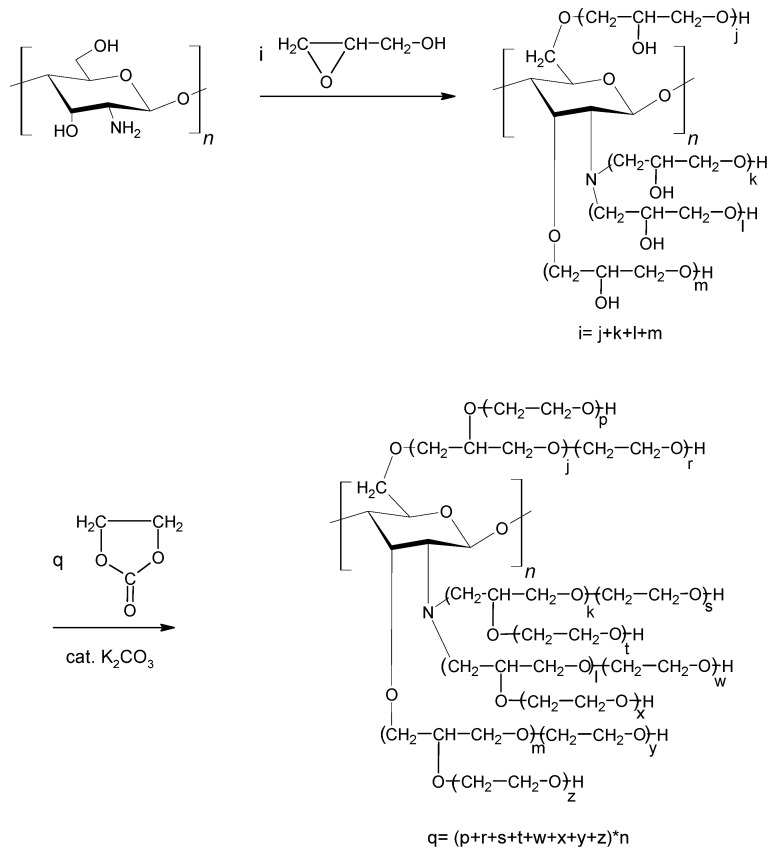
Hydroxyalkylation CS_O_ with GL and EC.

**Scheme 3:**
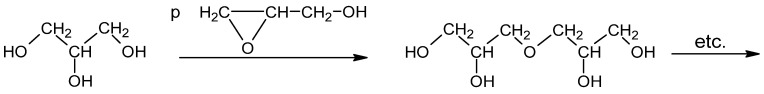
Hydroxyalkylation GLYC with GL.

CSs with high molecular weights are not readily available substrates to obtain polyols. However, we previously described the methods of the polyol synthesis from a water-soluble CS_WS_ [33]. Here, we replaced a chitosan with an oligomeric CSo which dissolves in water, although worse than the previously used CS_WS_. The reason for the lower water solubility of the CSo is a lower degree of deacetylation (DD = 68%) compared to that of the CS_WS_ (DD = 85%) [33]. The molecular mass of the CS_O_ was 8710 Da determined by the viscosimetric method. It seemed that this oligomer was a convenient substrate for hydroxyalkylation with GL, similar to the CS_WS_. The product of glycidylation was a semisolid resin; we have further converted it by the reaction with EC to obtain a liquefied product (Scheme 2). We tested three variants of the polyol synthesis. Firstly, we mixed the CS_O_ with GLYC in order to partially dissolve the CS_O_ and later added GL. The reaction was exothermic already upon the initial heating to 155 °C. After the consumption of GL, the semi-product was further reacted with EC in the presence of K_2_CO_3_ catalyst. The obtained polyol was liquid and contained the products of GLYC glycidylation (Scheme 3). In the second method, the straightforward reaction of the CS_O_ with GL in the absence of GLYC was attempted. The semi-product was again hydroxyalkylated further with EC. In the third method, the CS_O_ was mixed both with GL and EC. In the initial step of the process, EC acted as a solvent, and GL was a primary reagent, while further addition of K_2_CO_3_ enabled EC to react to complete the process.

**Figure 1:**
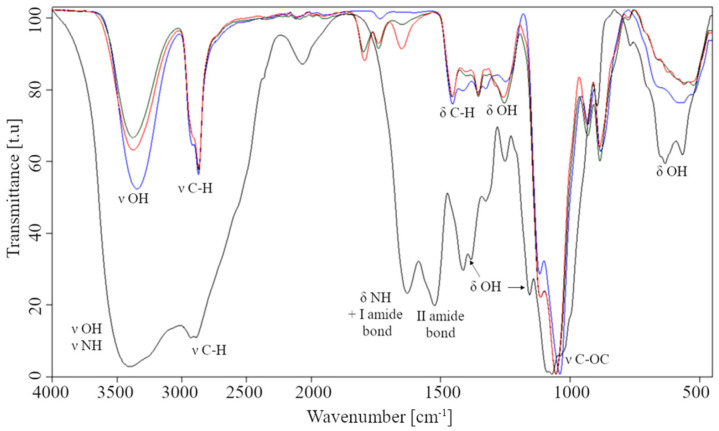
IR spectra of CS_O_ (black) and polyols (CS_O_ + GLYC + GL) + EC (blue), (CS_O_ + GL) + EC (green), and (CS_O_ + GL + EC) (red).

**Scheme 4:**
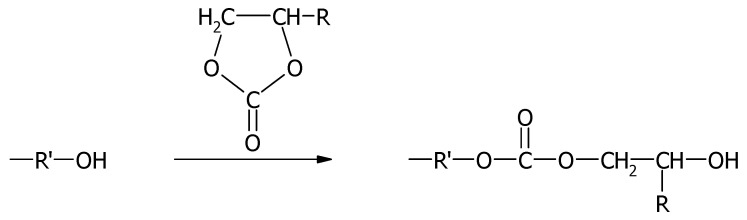
Incorporation of ester groups into polyol.

The reaction was monitored using IR and ^1^H-NMR spectroscopies, and the products were analyzed using the MALDI–ToF technique. Based on the IR and H-NMR spectra of polyols and the oligomeric chitosan, the functional groups of the chitosan involved in hydroxyalkylation were identified, and a polyol structure was suggested. The IR spectra of the obtained polyols were compared with those of the CS_O_. In the IR spectrum of the CS_O_ (Figure 1), the broad band centered at the 3380 cm^−1^ origin of the hydroxyl and the amine groups’ stretching vibrations, similar to the deformation vibrations of these groups at 1330 cm^−1^ and 1500 cm^−1^, respectively. The valence band of ether C-O-C was observed at ca. 1070 cm^−1^. The presence of non-deacetylated groups was manifested by amide bands I and II at 1629 cm^−1^ and 1524 cm^−1^, respectively. The latter overlapped with deformation band of the CS_O_ amine groups. In the IR spectra of polyols (Figure 1), a slight increase in the intensity of the band centered at 1030 cm^−1^ was observed compared to that of the CS_O_. This indicates the formation of additional C-O-C bonds. Moreover, the methine and methylene bands were found at 2900 cm^−1^ and within 1400–1300 cm^−1^ due to the ring opening of GL and EC and introducing them into the polyol structure. In the IR spectra of polyols, the valence and deformation bands of the hydroxyl groups were present at 3400 cm^−1^ and 1330 i 600 cm^−1^, respectively. In the spectrum of the (CS_O_ + GL + EC) polyol, a low intensity band was observed at 1780 cm^−1^ and attributed to the valence vibration of the carbonyl group (Figure 1), which suggested the presence of the ester groups in a polyol, which may form upon the EC ring opening according to Scheme 4.

**Figure 2:**
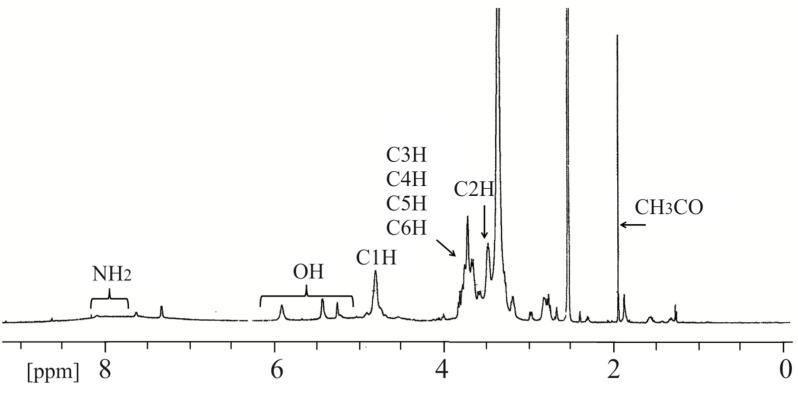
H-NMR spectrum of CS_O_.

**Figure 3:**
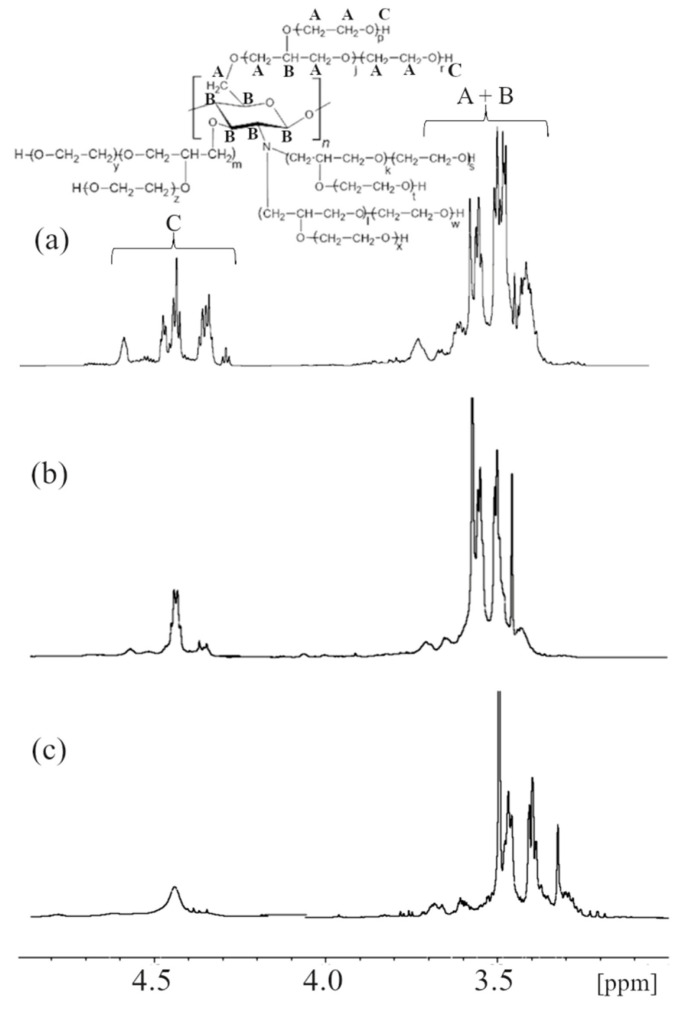
H-NMR spectra of polyols (CS_O_ + GLYC + GL) + EC (**a**), (CS_O_ + GL) + EC (**b**), and (CS_O_ + GL + EC) (**c**).

In the ^1^H-NMR spectrum of the CSo (Figure 2), the amine group protons showed broad resonance within 7.0–9.0 ppm, while the primary and secondary hydroxyl proton resonances were present within 5.2–5.9 ppm. The C_1_-H resonance was found at 4.55 ppm, while the resonances from the methine protons attached to C_3_–C_6_ were located within the 3.3–3.7 ppm region. The C_2_H resonance was partially overlapped with residual H_2_O at 3.4 ppm. The methyl group resonance from the non-deacetylated CS_O_ was present at 1.7 ppm [11]. The amine proton resonances were absent in the spectra of the obtained polyols, which was expected due to the reaction of the amine groups with the hydroxyalkylating agents. The proton resonances within 3.3–3.7 ppm showed a changed pattern in the spectra of polyols (Figure 3). Some additional resonances attributed to the methine and methylene groups appeared in that region, indicating the GL and EC ring opening upon the hydroxyalkylation of the CS_O_. Moreover, the resonances from the hydroxyl protons previously observed in the spectrum of the CSo (5.2–5.9 ppm) disappeared in the spectra of polyols, while other hydroxyl proton resonances appeared within 4.4–4.6 ppm as a consequence of the GL and EC ring opening. No considerable differences in the NMR spectra of various polyols were found, indicating that the structure of polyols is very similar.

**Scheme 5:**
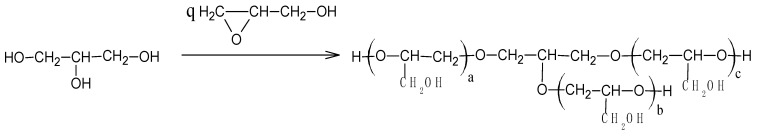
Reactions of glycerol with GL, where a + b + c = q.

**Scheme 6:**
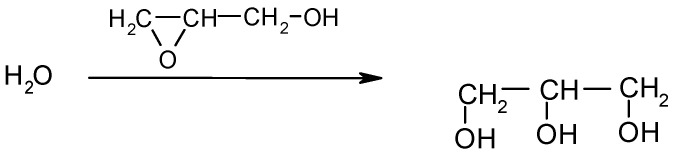
Formation of GLYC during synthesis of polyols.

**Scheme 7:**
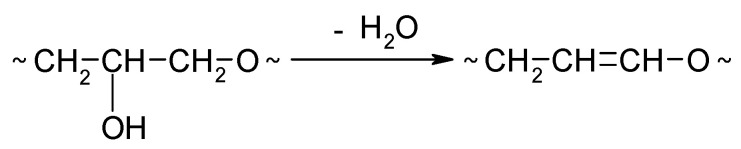
Dehydration of semi-products at a high temperature.

The side-products accompanying polyols were identified using the MALDI–ToF spectrometry (Table 1, Tables S1 and S2). In all cases, the peaks from GL and GLYC were observed (Table 1, entries 2, 3; Tables S1 and S2, entries 1, 3, 4). In the spectrum of the (CS + GLYC + GL) + EC polyol, the peaks indicating the presence of the GLYC hydroxyalkylation with glycidol were found (Scheme 5) (Table 1, entries 6, 12). These peaks were also present in the spectra of the (CS + GL) + EC and (CS + GL + EC) polyols (Table S1, entries 8, 9, 11; Table S2, entry 6), which were obtained without the participation of GLYC. The presence of GLYC is a consequence of the reaction of GL with water (Scheme 6), which is formed upon the dehydration of the semi-products at elevated temperatures (Scheme 7).

**Scheme 8:**
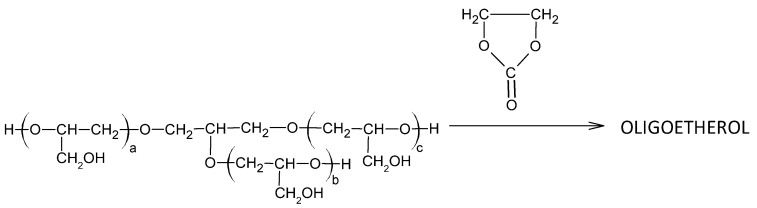
Reaction of semi-product with EC.

A semi-product of hydroxyalkylation with GL can further react with EC (Scheme 8) (Table S1, entries 12, 13, 15; Table S2, entries 7, 8 10).

**Scheme 9:**
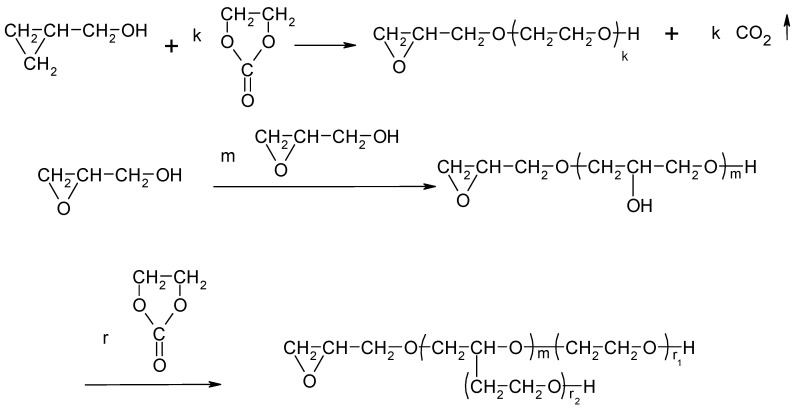
Reaction EC with GL and its oligomers, where r = r_1_ = m r_2_.

The products of GL and its oligomers’ hydroxyalkylation with EC (accompanied with the CO_2_ release) were present in all polyols (Scheme 9; Table 1, entry 5; Table S1, entries 5, 6, 7, 16, 17; Table S2, entries 5, 11). The side-products can also undergo dehydration in reaction conditions applied here (Scheme 7; Table 1, entries 1, 7, 10, 14; Table S1, entries 2, 10, 14, 17; Table S2, entries 2, 9, 12, 13). We concluded that using the MALDI–TOF spectrometry, we identified the products of the oligomerization of GL and the presence of the products of the hydroxyalkylation of glycerol with glycidol and then with EC.

**Scheme 10:**
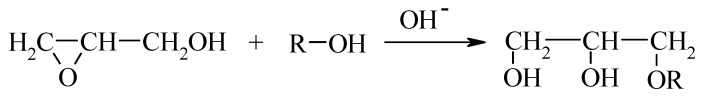
Increase in product functionality after reaction with GL.

The obtained polyols were dark brown, viscous resins. The hydroxyl number (HN) and some physical parameters such as density, viscosity, and surface tension of the obtained polyols were determined (Table 2). The HN of polyols fell within 482–548 mg KOH/g, which suggests their applicability for obtaining rigid polyurethane foams. The obtained HN values evidenced the high functionality of the polyols, which is presumably due to the use of GL as a hydroxyalkylating agent, because every added amount of GL to the hydroxyl group of the CS_O_ increased the functionality of the polyols by 1 (Scheme 10).

Therefore, the products of the reaction between the CSo and GL were semisolid resins not miscible with isocyanates. The character of these resin products is consistent with the plenty of hydrogen bonds present in the structure. When oxyalkylene chains undergo elongation upon reaction with EC, the number of hydrogen bonds decreases, and eventually, the resins show lower viscosity. The densities and surface tensions of polyols are similar and fall within the region of 1.262–1.275 g/cm^3^ and 38.1–38.6 mN/m, respectively. The lowest viscosity was obtained from the polyol produced according to method 3, i.e., the all-reagent method. Presumably, the low viscosity of the product is due to the shortest time of reaction (compare Section 2.2). In other cases, the prolonged heating led to the partial condensation of the hydroxyl groups and eventually increased the viscosity of the product. Typical dependences of physical parameters on the temperature were observed for the obtained polyols (Figure S1). They fell within the region suitable for polyols used for the synthesis of PUFs. The polyols suitable for obtaining PUFs have the viscosity within 200–30,000 mPas [45]. According to our experience, the surface tension of polyols suitable for PUFs should be within 30–50 mN/s. Low surface tension is a preferable property of the polyol because it enables effective foaming.

### 3.2. Analysis of Reaction between CS with GL and EC

**Figure 4:**
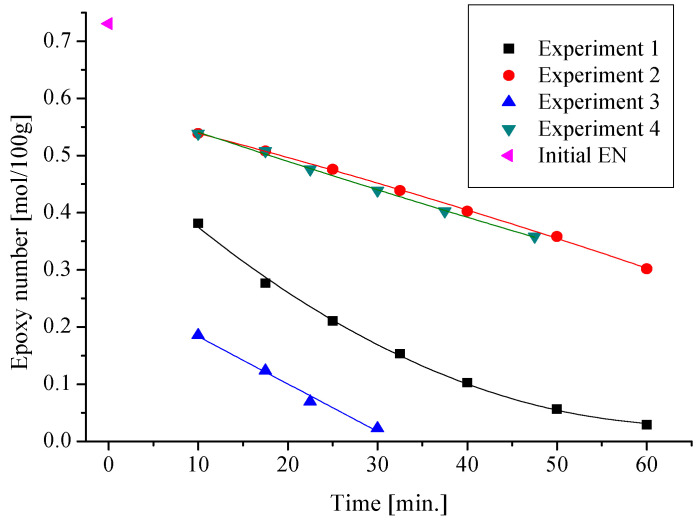
Changes in GL as a function of time in the CS_O_-GLYC-GL (experiment 1), cyclohexanone-GLYC-GL (experiment 2), CS_WS_-GLYC-GL (experiment 3), and cyclohexanone-GLYC-GL (experiment 4) systems at 130 °C.

The reactions of the CS_O_ with GL and EC has not been described in the literature until now. The polyols obtained in such reaction can be used to produce PUFs (vide infra). Therefore, the reaction of hydroxyalkylation was studied here in details. The system of reactants was chosen so that the mass ratio of the substrates was analogous to that in the described syntheses of polyols (see Section 2 and Section 2.2). The reaction in GLYC was executed with 1.2 g CS_O_, 9 g GLYC, and 12 g GL (experiment 1). The mixture was heated to 160 °C within 10 min in order to dissolve the CS_O_, and then the temperature was decreased to 130 °C. At this temperature, the consecutive consumption of GL could be monitored by the determination of EN. Then, the CSo was replaced with an inert substance, not reacting with GL, for which cyclohexanone was chosen, and again, the EN was determined to follow the consumption of GL (experiment 2). A fast decrease in the EN was observed for the system with the CS_O_ (Figure 4). The amount of GL (the epoxide groups) after dissolving the CS_O_ in GLYC and increasing the temperature to 130 °C (ca. 10 min) decreased from 0.7305 mol/100 g (initial EN) to 0.3817 mol/100 g, which corresponded to the 47.7% reaction of GL, while in the presence of cyclohexanone, the degree of the GL reaction was 0.5385 mol/100 g, which corresponded to the 26.3% consumption. The reaction of the CS_O_ with GL in GLYC ended after ca. 60 min (EN = 0.00); at the same time period, in the system without the CS_O_, only 58.9% of GL reacted with GLYC (EN = 0.3020). This indicated that GL reacted both with the CS_O_ and GLYC, which corroborated the analytical data obtained using the MALDI–ToF technique (Table 1).

For comparison, the same reactions were monitored using the methods previously employed in a study of the CS_WS_ and GL in GLYC [33] (Figure 4, experiments 3 and 4). In this case, the amount of GL after dissolving the CS_WS_ and increasing the temperature to 130 °C (10 min) diminished to 0.1858 mol/100 g, which corresponded to the 74.6% GL consumption, while in absence of the CS_WS_, the consumption was 26.3%. The reaction of the CS_WS_ with GL in GLYC took ca. 30 min at a temperature of 130 °C; at the same time, in the presence of inert cyclohexanone, instead of the CS only 40% of GL reacted with GLYC (EN = 0.4388 mol/100 g). Based on the numbers obtained in these experiments, we can conclude that the CS_WS_ was more reactive towards GL compared to the CSo. It was an expected result due to the higher percentage of the amine groups in CS_WS_ (DD = 85%) compared to the CSo (DD= 68%).

**Figure 5:**
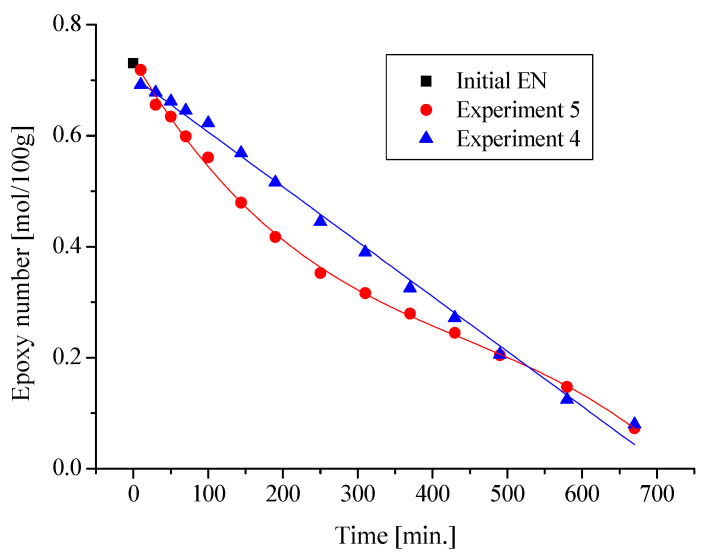
Changes in GL as a function of time in the CS_WS_-H_2_O-GL (experiment 5) and dioxane-H_2_O-GL (experiment 6) systems at 90 °C.

The reaction between the CS_WS_ and GL in water, described previously in [33], was different (Figure 5).

The experiment was performed as follows: 12 g GL was added to 1.2 g CS_WS_ in 9 g water, and the mixture was heated to 90 °C for 10 min. At this temperature, the first measurement was made, and then the changes in the GL content over time were studied (experiment 5). For comparison, the same protocol was used with dioxane instead of the CS_WS_ (experiment 6). Faster GL consumption was observed in water compared to GLYC. As it has been mentioned previously [33], the amount of distilled water after the completion of reaction corresponded to the amount of water left in the reaction mixture, assuming that GL reacts completely only with water but not with the CS_WS_. Thus, the fast consumption of GL is not due to the reaction with the CS_WS_ but to the presence of the amine groups in the structure of the CS_WS_ merely, which catalyzed the reaction between water and GL (compare with Scheme 6). Such catalysis by the amines in the hydroxyalkylation reactions with epoxide is known [46].

Based on the results, we can conclude that GLYC promotes the hydroxyalkylation of chitosan better compared to water. In the latter case, the CS_WS_ remains dissolved in the oligomer obtained by the reaction of GL with water. The hydroxyalkylation of the CS_WS_ occurs in the next step of the reaction with EC [33].

The reason for the prevailing hydroxyalkylation of the CS in GLYC vs. water is the lower amount of GLYC (0.13 mol) compared to that of water (0.5 mol) in the reaction mixture, even if we take into account the contribution of the three hydroxyl groups in the GLYC (0.39 mol). Moreover, the viscosity of the mixture plays its role. It is much lower in water, which in turn increases the mobility of the CS_WS_ and GL molecules and the probability of their collisions.

**Figure 6:**
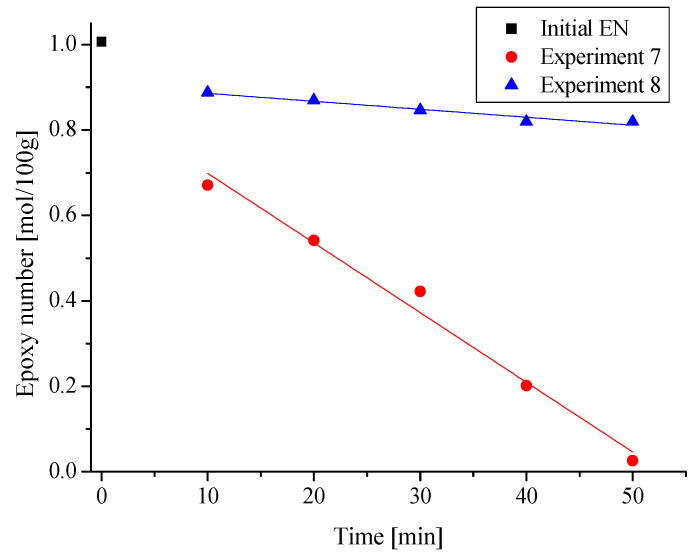
Changes in GL as a function of time in the CS_O_-GL (experiment 7) and cyclohexanone-GL (experiment 8) systems at 130 °C.

We also studied the reaction between the CS_O_ and GL in the absence of a solvent (Figure 6, experiments 7 and 8) according to synthesis 2. The mass proportions of reagents were the same as in the synthetic protocol, namely 4 g CS_O_ and 15 g GL, and in the control experiment with cyclohexanone instead of the CS_O_. We followed the consumption of epoxides. The control experiment was aimed at investigating the possibility of the oligomerization of the GL itself. The starting point was taken after dissolving the CSo at 130 °C (10 min). Immediately after mixing the reagents, the EN was 1.0067 mol/100 g, while after reaching the 130 °C temperature, it dropped to 0.6711 mol/100 g, corresponding to 33.4% GL consumption. The full consumption of GL was achieved after 50 min. In the same time scale, the reference sample showed the EN equal to 0.8879 and 0.8195 mol/100 g, which corresponded to the 11.8% and 18.6% GL consumption. The result indicated that the oligomerization of GL occurs simultaneously with the CS_O_ hydroxyalkylation, although to a small extent (see Scheme 5), which corroborated the MALDI–ToF results (Table S1).

**Figure 7:**
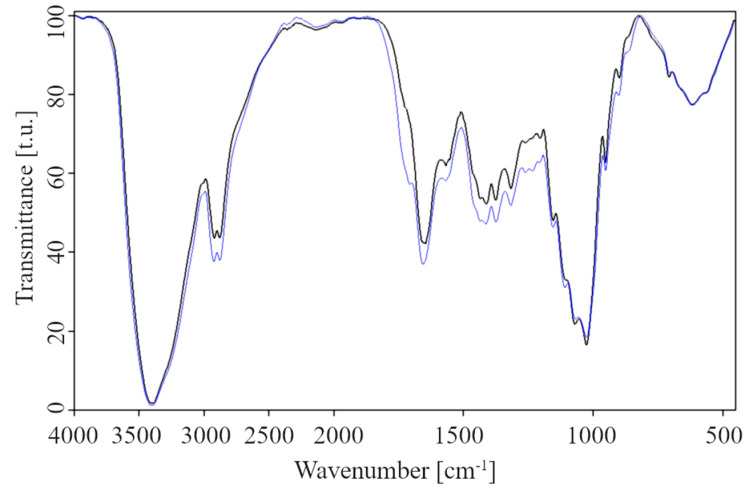
IR spectra of CSo:GL = 1:1 (black) and CSo:GL = 1:2 (blue).

**Figure 8:**
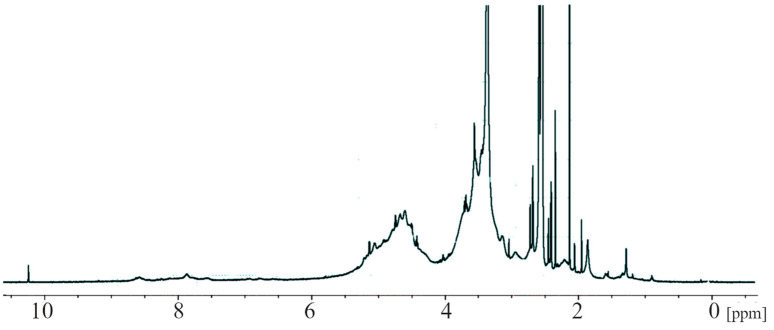
H-NMR spectrum of CSo:GL = 1:1.

The reaction of the CS_O_ with GL at the initial molar stoichiometry 1:1 was monitored in DMSO because the CS_O_ did not dissolve completely in GL. The reaction was performed at 130 °C, and the product was isolated and analyzed using the IR and HNMR spectroscopies. The IR spectrum of the semi-product CS_O_:GL = 1:1 (Figure 7) compared to that of the CS_O_ (Figure 1) showed the change in the hydroxyl-bond valence pattern at 3400 cm^−1^. The intensity of the amine deformation band at 1520 cm^−1^ decreased significantly, which indicated the reaction of GL with the amine groups. The high reactivity of the amine groups against the GL ring was attributed to the higher nucleophilicity of the amine groups compared to that of the hydroxyl. In the spectrum of the consecutive semi-product obtained from CS_O_:GL = 1:2, the reaction did not show any additional changes. Based on that, we can conclude that a further reaction took place between the hydroxyl groups of the substrate and the semi-product with GL. It was confirmed by the H-NMR spectrum of the semi-product of CS_O_:GL = 1:1 (Figure 8). The disappearance of the broad band at 7.0–9.0 ppm for the amine groups, which was present in the spectrum of the CS_O_, and other O-H resonances in the range of 5.2–6.0 ppm, and the simultaneous growing of resonances at 4.1–5.1 ppm was observed, as identified by the selective deuteration with D_2_O. Moreover, in the spectrum of the CS_O_:GL = 1:1 semi-product, there were no resonances from GL at 2.7 and 3.0 ppm [47], which are characteristic for the GL spectrum. Upon the reaction, the shape of the resonances at 3.2–3.5 ppm changed, and new resonances from methine and methylene protons grew, indicating the GL ring opening.

**Figure 9:**
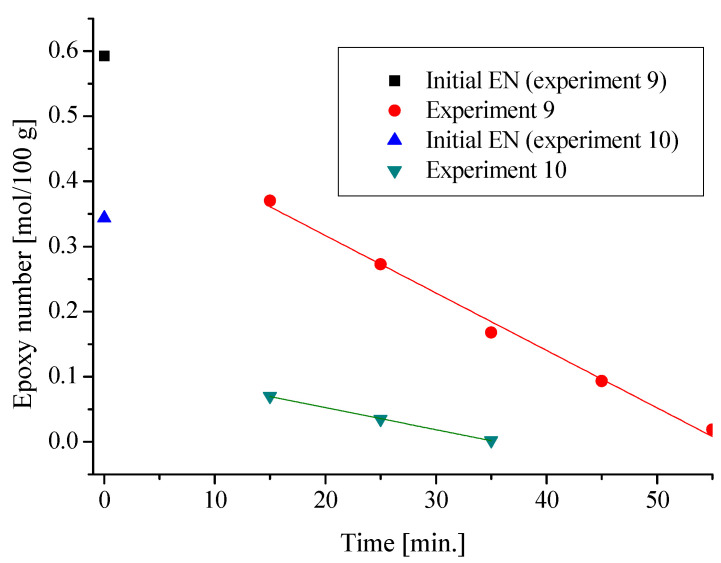
Changes in GL as a function of time in the CS_WS_-GL-EC (experiment 9) and CS_O_-GL-EC (experiment 10) systems at 130 °C.

According to synthesis 3, all the three reagents, CS, GL, and EC, were mixed at the following ratio: 1.2 g CS_WS_ + 15 g GL + 18 g EC (initial EN = 0.5927 mol/100 g) or 1.2 g CSo + 4.5 g GL + 12 g EC (initial EN = 0.3436 mol/100 g). The EC was the solvent for that process. The components were heated analogously to synthesis 3 to 190 °C for 15 min to completely dissolve the chitosan and determine the EN (Figure 9). In this condition, 37.5% of GL was consumed upon the reaction with the CS_WS_, while the 79.6% GL reacted with the CSo. The differences between the two reaction systems depend on the amount of GL vs. CS and the molecular mass of the latter. The chitosan of a lower molecular mass (CSo) dissolved faster, had lower viscosity, and greater mobility of the molecules, which makes them more likely to collide with the GL molecules. The higher molecular mass and viscosity of the CS_WS_ imposed a slower reaction. After the reaction with GL was complete, the amount of EC in the post-reaction mixture was monitored to answer the question whether it was already involved in the hydroxyalkylation in the absence of K_2_CO_3_. It was found that in the case of the system with the CS_WS_, the percentage of the EC drops from 52.6% after mixing the components to 48.5% after the total consumption of GL (40 min). In the case of the CS_O_, this parameters were 67.8% and 63.2% (after 20 min). Thus, in both cases EC reacted to a small extent. The reaction with EC was further triggered by the addition of the catalytic amount of K_2_CO_3_.

The analysis of the reaction of chitosan in various environments (water, GLYC, or GL) allowed us to determine the reactivity of the substrates and understand the reaction mechanism. Understanding the reaction mechanism based on the reactivity is important to optimize the synthetic reaction conditions, namely the temperature, concentration of the reagents and catalyst. This can further help to design and optimize the reactors in a scaled-up process.

### 3.3. Polyurethane Foams

Synthesized polyols were used to obtain PUFs on a laboratory scale. The kind of the polyol, the amount of isocyanate (pMDI), the catalyst, and the foaming agent were varied to optimize the PUFs. Gaseous CO_2_ was used as a foaming agent in the reactions of water with pMDI. The optimized compositions are shown in Table 3. The cream and rise times for all compositions were similar and fall within 41–44 s and 33–43 s, respectively. They depended slightly on the kind of the polyol and the amount of the catalyst. Exceptional was the PUF obtained from the (CS_O_ + GL + EC) polyol, which had shorter cream and rise times: 38–40 s and 30–39 s, respectively. This can be attributed to the presence of a large number of reactive hydroxyl groups in the (CS_O_ + GL + EC) polyol, as determined by HN = 510 mg KOH/g. Generally, high hydroxyl numbers also result in a short drying time for the composition. The PUFs were optimized in terms of the amount of isocyanate. It was found that the best PUFs were obtained at mixtures with a molar ration of the isocyanate groups to hydroxyl groups (isocyanate coefficient) of 1.0–1.3 (Table 3).

**Figure 10:**
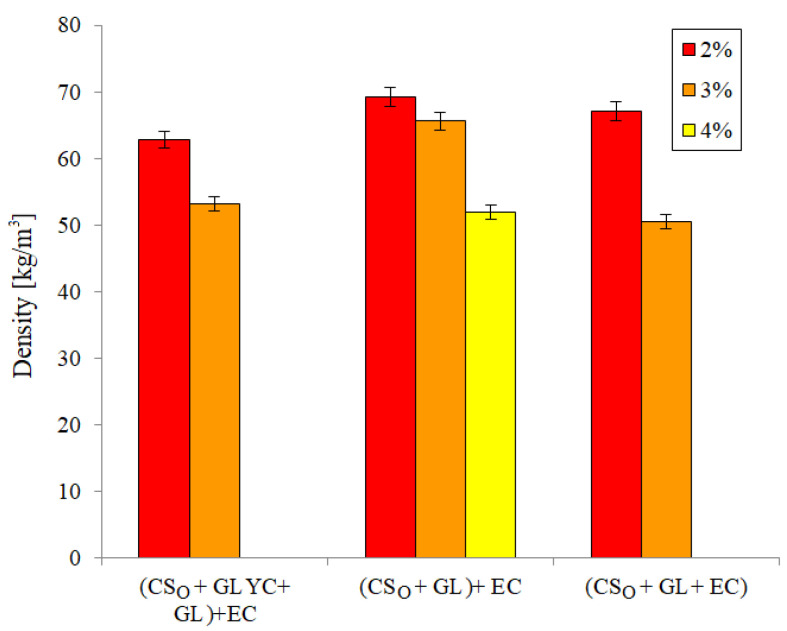
The density of the obtained polyurethane foams (determined with 2.1% accuracy). The water content in the foaming composition per 100 g of polyol is shown in the insert.

Crucial properties of PUFs were determined, such as apparent density, volume water uptake, dimension stability, thermal conductivity coefficient, pore size, thermal resistance, compressive strength, and glass transition temperature. All these parameters are relevant for the quality and functionality of PUFs, depending on their type and application. One of these parameters of PUFs is, for instance, their apparent density. It also influences other physical and mechanical properties such as compressive strength. The apparent density of PUFs is illustrated in Figure 10.

**Figure 11:**
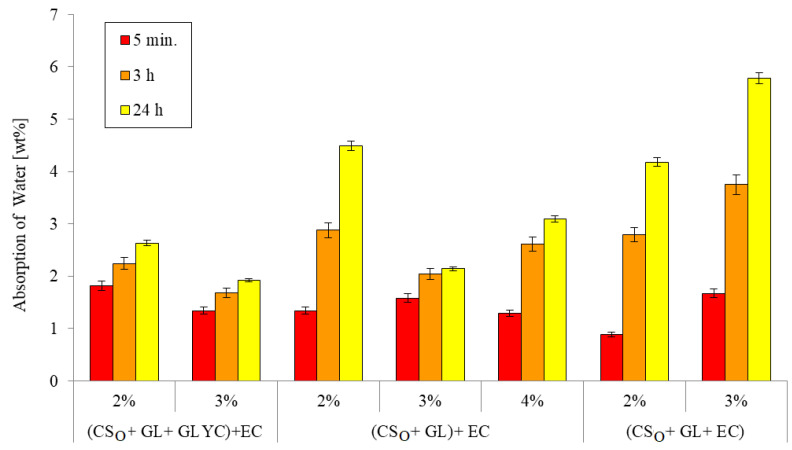
Absorption of water in the obtained polyurethane foams (determined with ±1.2% accuracy). The appropriate shade of color indicates the time the foam stayed in the water (shown in the insert). The water content in the foaming composition per 100 g of polyol is given below the horizontal axis of the graph.

The apparent density of PUFs was dependent on the amount of water in the foaming composition. At a higher limit (3% water), more CO_2_ was evolved, resulting in better foaming, and eventually, low-density PUFs were formed, with a 51–64 kg/m^3^ value. At a lower water limit (2%), the apparent density of PUFs was 63–69 kg/m^3^. Further increase in water in the foaming mixtures led to the deterioration of the mechanical resistance of PUFs and irregular, large pores. The exception was the PUF obtained from the (CS_O_ + GL) + EC polyol and 4% water, which had an apparent density of 52 kg/m^3^ and high compressive strength (vide infra). The values of the apparent density of the obtained PUF correspond to those of rigid PUFs. The water uptake after the 24 h exposition is low and fall within 1.9–4.6%, except for the PUF obtained from the (CS_O_ + GL + EC) polyol, for which the uptake was 5.8% (Figure 11). Low water uptake of PUFs is preferable, because it allows for the application of such PUFs as isolating materials in humid surroundings without the loss of their isolation capability [48]. The water uptake of the PUFs used in the building construction industry should be within 3–5%. Such conditions are fulfilled by the PUFs described here. Low water uptake values suggest the presence of closed pores in the PUFs, which is an advantageous property of the PUFs used as thermal isolation materials.

**Figure 12:**
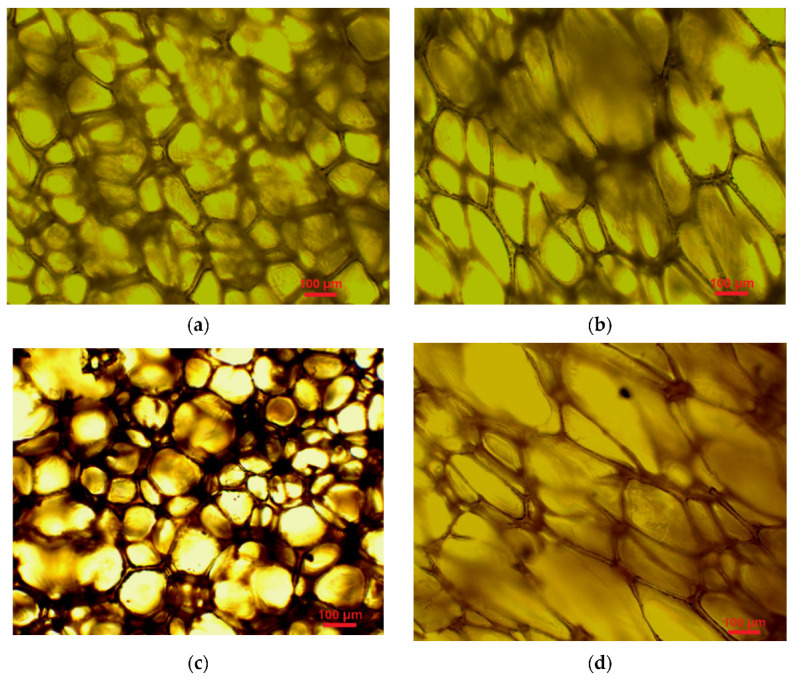

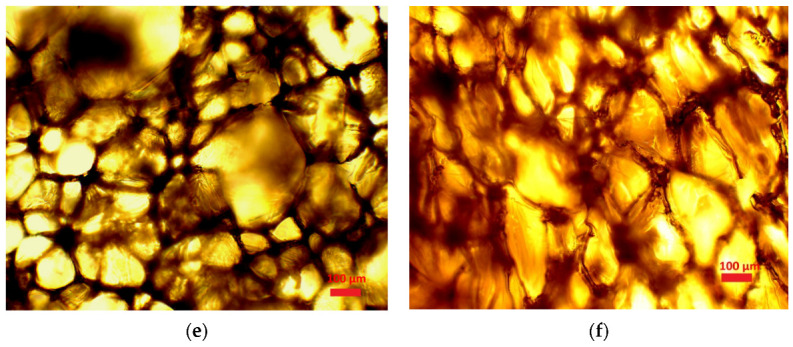
Optical microscopy images of foam obtained from polyols (CSo + GL + GLYC) + EC (**a**,**b**), (CSo + GL) + EC (**c**,**d**), and (CS_O_ + GL + EC) (**e**,**f**) with 2% (**a**,**c**,**e**), 3% (**b**,**f**), and 4% (**d**) water content/100 g of polyol.

The pores of the PUFs obtained from the (CS_o_ + GLY + GL) + EC and (CS_o_ + GL) + EC polyols and 2% water were close to spherical (Table 4, Figure 12a,c) with the longest diameter of 129–148 µm and the shortest diameter of 95–123 µm. The increase in the water content to 3% or 4% in the foaming composition resulted in considerable elongation of the pores (Table 4, Figure 12b,d). In such cases, the longest diameter was within 109–436 µm, while the shortest diameter was within 71–160 µm. This explained why the compressive strength of the PUFs obtained with the 3 and 4% water in the foaming composition was lower. The thickness of the pore walls was between 12 and 18 µm. The PUFs obtained from the (CS_o_ + GL + EC) polyol had oval pores, independently of the amount of the foaming agent; the pores obtained with the 3% water were smaller. This can be attributed to the fast crosslinking related to the high values of HN of the used polyol. Moreover, a known relationship was noticed: the PUFs with smaller and oval pores showed a higher compressive strength compared to that of the PUFs with the elongated pores.

**Figure 13:**
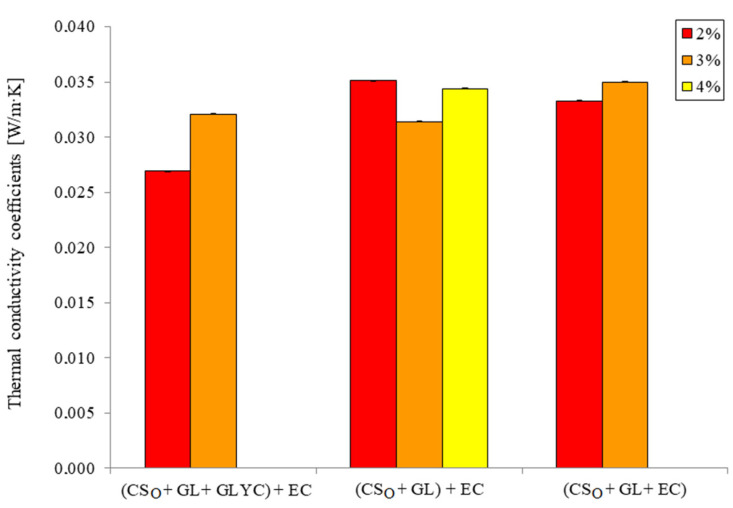
Thermal conductivity coefficients of obtained polyurethane foams (determined with 0.1% accuracy). The appropriate shade of color indicates the water content in the foaming composition per 100 g of polyol (in the insert).

In the case of rigid PUFs, the thermal conductivity coefficient is usually within the 0.020–0.035 W/m·K region. The thermal conductivity coefficient of the PUF obtained from (CS_O_ + GLYC + GL) + EC and 2% foaming agent was typical of rigid PUFs (0.0269 W/m·K) [45]. For other PUFs, it fell within 0.0314–0.0351 W/m·K (Figure 13). The thermal conductivity coefficients (λ) depends on amount of open and closed pores in foams. Generally, more open pores result in low λ. Such situation was observed in the studied PUFs. The increase in the open pores measured by the water uptake corroborated the increase in the thermal conductivity coefficients.

The PUFs had good dimensional stability. The thermal exposure of PUFs for 40 h at 150 °C caused distortions within (−5.56)–(+2.04)%. Typically, the acceptable dimension stability of polyurethane foams is 3–5%. In some cases, the shrinkage was observed in one direction and elongation in another (Table 5).

**Figure 14:**
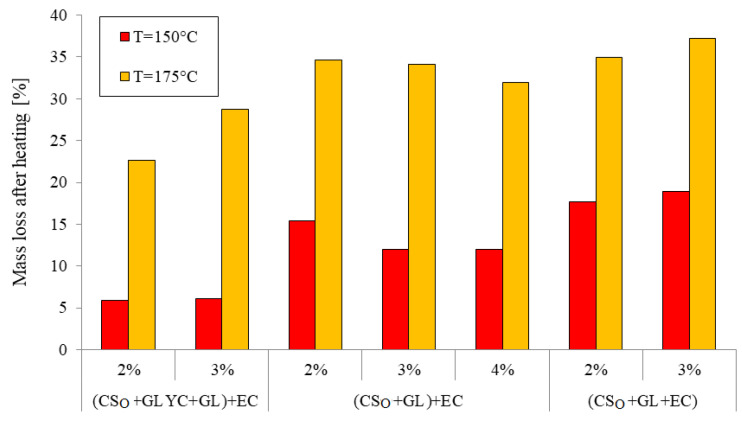
Mass loss of PUFs after exposure for one month at 150 °C and 175 °C.

The thermal resistance of the PUFs was analyzed by the mass loss during one-month thermal exposure of the PUF samples at 150 and 175 °C with the concomitant measuring of compressive strength before and after the thermal exposure. Thermal resistance means that a material maintains the physical and mechanical properties at the testing temperature. Generally, the mass loss can be related to physical conversions, such as evaporation of TEA or water, and chemical degradation leading to volatile side-products. The PUFs obtained here were rigid at room temperature, and they remained as such after the thermal exposure. The PUFs’ mass loss is the largest in the first day of the exposure. When heated to 150 °C, the mass loss was within 12–19% (Figure 14) in the case of the PUFs obtained from the (CS_O_ + GL) + EC or (CS_O_ + GL + EC) polyols, i.e., slightly larger compared to that of the PUFs obtained from the CS_WS_ in the same conditions (10.4–17.4%) [33]. The lowest mass loss was observed for the PUFs synthesized in GLYC (5.9–6.1%). It was comparable to that of the thermoresistant PUFs obtained from the polyols containing azacyclic components such as isocyanuric acid and purine (6–9%) [49]. The PUFs based on the (CS_O_ + GLYC + GL) + EC polyol showed the highest thermal resistance also at 175 °C (Figure 14). Their mass loss after the one-month thermal exposure was 22.6–28.7%, while in other PUFs, it was 6–10% higher. Such a high thermal stability of these PUFs can be related to the presence of the multifunctional oligomeric products of glycerol with GL during the synthesis of (CS_O_ + GLYC + GL) + EC polyol. These oligomeric side-products can be responsible for additional crosslinking reactions.

**Figure 15:**
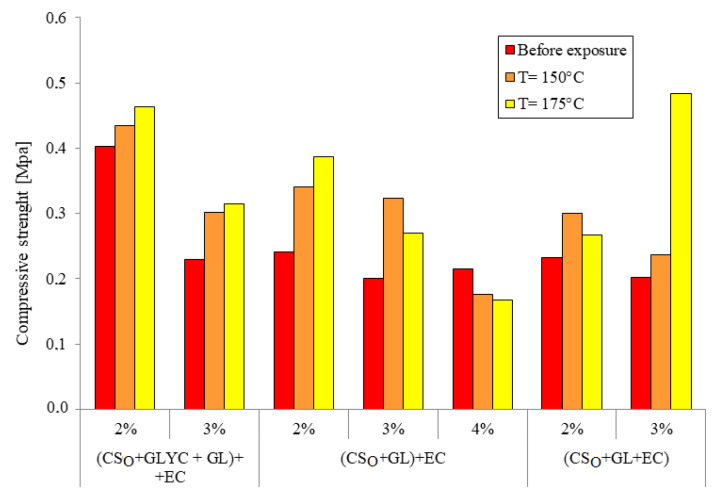
Compressive strength of PUFs before and after exposure for one month at 150 °C and 175 °C.

The compressive strength of the obtained PUFs is typical of rigid PUFs (0.200–0.241 MPa, Figure 15).

In the case of the PUF obtained from the GLYC solution and 2% water, the compressive strength was twice as high (0.403 MPa). This can be explained by the additional crosslinking and spherical pores (Figure 12a). In other cases, the kind of the polyol used did not influence the compressive strength. It was a little lower for the PUFs obtained with a larger amount of foaming water (3% and 4% water/100 g polyol) due to a lower apparent density (Figure 10) and larger and elongated pores (Table 4). The PUFs showed a greater compressive strength after the thermal exposure at 150 °C or 175 °C compared to that before the exposure. This can be explained by the additional crosslinking of the PUFs upon heating. It was especially pronounced in the case of the PUFs obtained from the (CS + GL) + EC and (CS + GL + EC) polyols and 2% water; in those cases, the compressive strength increased after the thermal exposure at 150 °C and 175 °C by 41.1 and 28.8% and 60.6 and 14.6%, respectively. In some cases, the compressive strength decreased upon annealing at 175 °C compared to that upon annealing at 150 °C. Still, however, the compressive strength of the PUFs after the thermal exposure was larger compared to that of the untreated materials. The decrease in the compressive strength at 175 °C was probably related to the partial degradation of the samples. It is characteristic that the decrease in the compressive strength at 175 °C was incomparably low in relation to the loss of mass at this temperature. It should be remembered that the classic rigid PUFs are thermally resistant up to 90–110 °C, and above this limit, they lose their functional properties [1,2]. The PUFs described here were not designed as thermoresistant materials. The large mass loss at 175 °C was not surprising, because the degradation of PUFs starts in such condition, which is accompanied by graphitization. The latter is the reason for small changes in the compressive strength, which, according to our earlier experience, can even grow after annealing.

The obtained PUFs were subjected to a thermogravimetric analysis. The TG measurements confirmed the high thermal resistance of the PUFs obtained from the (CS_o_ + GLYC + GL) + EC polyol.

A 5% mass loss was observed at 221 °C and 247 °C for the PUFs obtained with 2 and 3% foaming water, respectively (Table S3). Some PUFs showed a 5% mass loss already at 75–87 °C. This is consistent with high moisture uptake from the air. This was also confirmed by the results of the water absorption determined in these foams (see Figure 11) and by the DSC analysis which evidenced the presence of volatiles in the PUFs (Figure S2).

**Figure 16:**
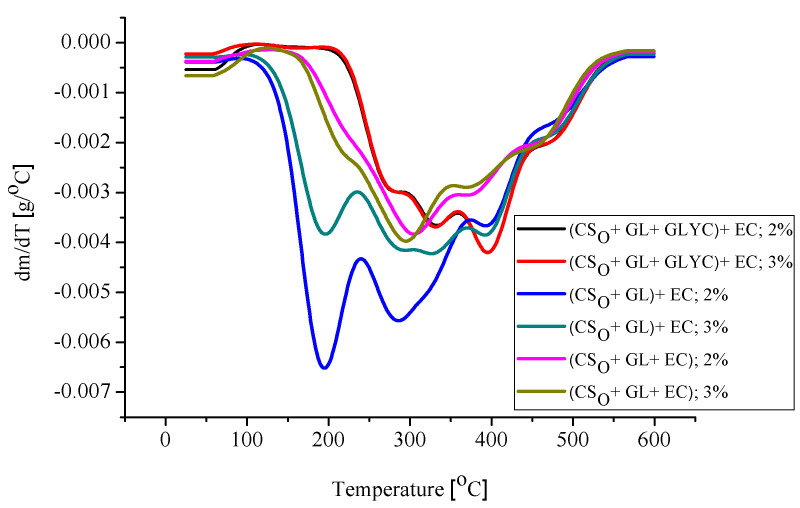
Thermal analysis of foams—differential mass change as a function of temperature (water content of 2% or 3% of the foaming compositions based on 100 g of polyol).

There were three peaks observed in the DTG curves of the PUFs, which were clearly pronounced at ca. 200, 280, and 400 °C (Figure 16). The first peak was due to the thermal dissociation of the urethane and urea bonds [1], the second one was due to the decomposition of the chitosan rings [11], while the third one was related to the decomposition of polyurethane to amine and carbon dioxide [1]. The total decomposition was completed at a temperature of ca. 600 °C. 

The DSC analysis of the PUFs evidenced the presence of volatiles in the unheated samples. The endothermic peak was observed within 15–110 °C in the first cycle, which was absent in the second cycle of the DSC profile (Figure S2). This was caused by the remnant TEA used as a catalyst and the moisture absorption from the atmosphere. The glass transition temperature could be determined from the second cycle and was found to be in the range of 72–113 °C (Table S3). The glass transition temperature allowed us to consider the materials as rigid PUFs.

The PUFs obtained here were compared with those obtained from the CS_WS_ [33]. The PUFs obtained from the CS_WS_ were synthesized from the polyols obtained in GLYC by the hydroxyalkylation with GL and EC, following a two-step protocol, in the reactions with GL and then EC and in the reactions, the environment of which was initially EC. The results are collected in Table 6. The PUFs obtained from the polyols obtained from the CS_O_ had a lower apparent density compared to that of the PUFs form the polyols obtained from the CS_WS_. This led to a slightly lower compressive strength of the former.

More favorable functional properties of the foams based on both chitosans were obtained for the products synthesized in the GLYC, because it leads to the high functionality of polyols. The best mechanical and thermal properties had the PUFS obtained from the (CSo + GLYC + GL) + EC polyols with 2% water. They had low apparent density, water uptake, and heat conductance coefficient. They also showed the lowest mass loss upon heating, while their compressive strengths increased upon the thermal exposure. In the case of other foams, we conclude that the chitosan-containing PUFs obtained from the water-soluble chitosan had better functional properties compared to those of the PUFs obtained from the oligomeric chitosan. Mainly, they had a higher thermal resistance measured by the mass loss, compressive strength, and water uptake. This is probably related to the higher molecular weight of the water-soluble chitosan, which translates into longer chains of the chitosan built into the PUF, rendering a more rigid and thermally resistant PUF. Thus, the best chitosan substrate for the PUF is the water-soluble chitosan due to its higher average molecular weight.

In summary, it can be stated that so far, the limitation of the use of chitosan for the production of polyurethane foams was the lack of appropriate solvents and difficulties in carrying out the chitosan hydroxyalkylation reaction with the use of oxiranes towards obtaining polyols. We found that glycerol and glycidol as hydroxyalkylating agents and then ethylene carbonate solved this problem in the case of the water-soluble chitosan.

Another problem that needs to be solved is the assessment of the possibility of using higher-molecular-weight chitosans for the production of polyols and polyurethane foams. We found here and in the previous studies that chitosan with a higher molecular weight could lead to PUFs of enhanced mechanical and higher thermal resistance. The synthesis of a polyol from a chitosan with a high molecular weight and GL as a reagent and a solvent was not possible due to insolubility of chitosan in such conditions, as was determined in the previous study [33]. However, it is possible to convert a high-molecular-weight chitosan into a polyol using a mixture of glycerol and glycidol as hydroxyalkylating agents and further hydroxyalkylation with EC to obtain a liquid polyol (similar to synthesis 2). The second method of the chitosan conversion into a polyol is based on preliminary dissolving the chitosan in a mixture of glycidol and ethylene carbonate and executing its hydroxyalkylation in the EC solvent. The second step is the further hydroxyalkylation of the product with the excess of EC at a higher temperature (similar to synthesis 3). These two synthesis routes were tested by us and made it possible to obtain polyurethane foams from the derived polyols. Detailed studies of the properties of the polyurethane foams based on chitosans with higher molecular weights will be conducted and published separately. These materials can find industrial applications due to their enhanced thermal resistance as thermal isolating materials for hot water or even overheated water vapor tubing.

## 4. Summary and Conclusions

1. Three methods for the synthesis of oligomeric polyols derived from chitosan were established.
2. The hydroxyalkylation of chitosan with glycidol in glycerol led to the formation of a multifunctional product, suitable for obtaining useful polyurethane foams.
3. The straightforward hydroxyalkylation of chitosan with glycidol was accompanied by the oligomerization of glycidol.
4. The hydroxyalkylation of chitosan with glycidol in the presence of ethylene carbonate was accompanied by minor hydroxyalkylation of the chitosan with ethylene carbonate.
5. The foams obtained from the polyols synthesized from the chitosan oligomer showed functional properties similar to those of the classic, rigid polyurethane foams, but they surpassed them in terms of heat resistance. They withstood long-term heating at 175 °C.
6. The polyurethane foams obtained from the polyols synthesized from the chitosan oligomer had advantageous properties such as the enhanced thermal resistance, dimensional stability, low water uptake, and high compression strength, growing remarkably upon the thermal exposure.

## Figures and Tables

**Table 1:** Interpretation of MALDI–TOF spectrum of polyol (CS + GLYC + GL) + EC.

Entry	Signal Position *m*/*z*	Relative Intensity of Signal (%)	Molecular Ion Structure	Calc. Molecular Weight (g/mol)
1	101.030	9.55	GL + OE – H_2_O + H^+^	101.060
2	112.894	43.94	GL + K^+^	113.000
3	114.873	27.36	GLYC + Na^+^	115.037
4	120.931	8.05	GL + OE + H^+^	119.071
5	140.940	3.97	GL + OE + Na^+^	141.053
6	196.884	66.10	GLYC + GL + CH_3_OH	198.110
7	249.102	7.78	3GL + OE – H_2_O + H^+^	249.134
8	293.124	9.67	GLYC + GL + 2OE + K^+^	293.100
9	323.133	3.79	GLYC + 2GL + OE + K^+^	323.111
10	367.196	10.48	4GL + 2OE – H_2_O + H^+^	367.197
11	393.999	100.00	5GL + Na^+^	393.174
12	411.176	13.76	GLYC + 3GL + Na^+^	411.184
13	441.177	8.28	GLYC + GL + 2OE + K^+^	441.174
14	590.816	12.02	GLYC + 5GL + 2OE + K^+^ 7GL + 2OE – H_2_O + H^+^	589.247 589.307

Description of abbreviations used in the table: GL—glycidol, GLYC—glycerol, OE—oxyethylene group from EC, H_2_O—water, K^+^—potassium ion from the catalyst K_2_CO_3_, Na^+^—sodium ion, CH_3_OH—methanol.

**Table 2:** Physical properties of polyols at 20 °C.

Type of Polyol	Density (g/cm^3^)	Viscosity (mPa∙s)	Surface Tension (mN/m)	Hydroxyl Number
(CS_O_ + GLYC + GL) + EC	1.2750	4268	38.6	548
(CS_O_ + GL) + EC	1.2615	4986	38.6	482
(CS_O_ + GL + EC)	1.2632	2807	38.1	510

**Table 3:** The influence of composition on the foaming process.

Polyol	Composition [g/100 g of Polyol)				Isocyanate Index	Foaming Process		
	pMDI	Water	Silicon L-6900	TEA		Cream Time (s)	Rise Time (s)	Tack-Free Time (s)
(CS_o_ + GLYC + GL) + EC	175	2	2.9	1.3	1.3	44	30	0
	180	3	3.9	1.2	1.3	44	43	8
(CS_O_ + GL) + EC	135	2	5.8	0.7	1.1	41	33	10
	155	3	4.8	0.9	1.2	43	39	10
(CS_O_ + GL + EC)	165	4	4.8	0.9	1.0	40	39	10
	155	2	5.8	0.5	1.0	38	30	9

**Table 4:** Results of micrograph analyses.

Foam Obtained from Polyol	Amount of Water/100 g of Polyol	Larger Diameter (µm)	Smaller Diameter (µm)	Thickness of Cell Wall
(CS_o_ + GLY + GL) + EC	2	129 ± 16	95 ± 12	15 ± 3
	3	342 ± 55	172 ± 34	14 ± 2
(CS_o_ + GL) + EC	2	148 ± 23	123 ± 20	19 ± 4
	3	109 ± 33	71 ± 8	12 ± 3
	4	436 ± 130	160 ± 44	14 ± 3
(CS_o_ + GL + EC)	2	265 ± 53	115 ± 25	18 ± 3
	3	192 ± 71	122 ± 19	18 ± 5

**Table 5:** Dimensional stability of foams.

Foam Obtained from	Water (%)	Dimensional Stability (%) at a Temperature of 150 °C					
		Length Change after		Width Change after		Height Change after	
		20 h	40 h	20 h	40 h	20 h	40 h
(CS_O_ + GLYC + GL) + EC	2	−0.50	−0.60	+0.74	+1.15	−0.86	−2.82
	3	+0.85	+1.35	+1.36	+0.24	+0.76	+2.04
(CS_O_ + GL) + EC	2	−2.74	−3.33	−2.06	−3.12	−0.88	−1.86
	3	−0.42	−1.29	−1.83	−4.19	−1.44	−2.40
	4	−0.16	+1.26	+5.02	+6.25	+0.69	+0.41
(CS_O_ + GL + EC)	2	−3.61	−3.90	−4.64	−5.56	+3.90	+1.66
	3	−2.94	−3.96	−3.59	−5.02	+1.01	−2.59

**Table 6:** Comparison of some properties of foams obtained with the participation of CS_WS_ and CS_O_.

Foam Obtained from	Water (%)	Density (kg/m^3^)	Absorption of Water (wt%) after			Heat Conductance Coefficient (W/m∙K)	Mass Loss in wt% after Exposure for Month at Temperature		Compressive Strength [MPa]		
									before Exposure	after Exposure at Temperature	
			5 min	3 h	24 h		150 °C	175 °C		150 °C	175 °C
(CS_WS_ + GLYC + GL) + EC	2	70.3	0.56	1.59	3.58	0.0231	17.4	34.6	0.513	0.694	0.791
	3	55.8	2.24	4.20	6.66	0.0245	10.4	24.2	0.337	0.510	0.238
(CS_O_ + GLYC + GL) + EC	2	63.0	1.85	2.20	2.6	0.0269	5.9	22.6	0.403	0.435	0.463
	3	52.0	1.40	1.60	1.90	0.0321	6.1	28.7	0.229	0.301	0.314
(CS_WS_ + GL) + EC	2	69.0	0.80	1.30	2.29	0.0366	14.9	27.7	0.119	0.271	0.373
	3	57.0	0.59	1.59	2.52	0.0356	14.1	28.5	0.203	0.235	0.219
(CS_O_ + GL) + EC	2	69.2	1.34	2.88	4.49	0.0351	15.4	34.6	0.241	0.340	0.387
	3	65.6	1.58	2.04	2.14	0.0314	12.0	34.1	0.200	0.323	0.270
(CS_WS_ + GL + EC)	2	73.1	0.45	1.67	2.47	0.0226	10.4	24.8	0.283	0.336	0.328
	3	56.0	0.74	1.52	2.46	0.0345	10.1	26.4	0.227	0.249	0.248
(CS_O_ + GL + EC)	2	67.1	0.89	2.79	4.18	0.0333	17.7	34.9	0.233	0.300	0.267
	3	50.5	1.67	3.75	5.78	0.0350	18.9	37.2	0.202	0.236	0.483

## Data Availability

Not applicable.

## Supplementary Materials

The following supporting information can be downloaded at https://www.mdpi.com/article/10.3390/polym15143084/s1. Figure S1: Changes of density (a), viscosity (b), and surface tension (c) of polyol as a function of temperature; Figure S2: Thermogram of foam obtained from polyol (CS + GL + EC) (2% H_2_O/100 g of polyol).; Table S1: Interpretation of MALDI-TOF spectrum of polyol (CS + GL) + EC; Table S2: Interpretation of MALDI-TOF spectrum of polyol (CS + GL + EC); Table S3: Thermal analysis of foam determined by the dynamic method.
