# Supplementary material for: Chitosan Oligomer as a Raw Material for Obtaining Polyurethane Foams
Source: Polymers (Basel). 2023 Jul 18;15(14):3084. doi: 10.3390/polym15143084 (PMC10384285; doi:10.3390/polym15143084)
Supplement: Supplementary file 1 [file polymers-15-03084-s001.zip › polymers-2498634-supplementary.pdf]

Supplementary Materials

Chitosan Oligomer as a Raw Material for Obtaining Polyurethane Foams

Anna Strzałka [1], Renata Lubczak [2] and Jacek Lubczak [2,*]

[1] Doctoral School of Engineering and Technical Sciences, Rzeszow University of Technology, Al. Powstancow Warszawy 6, 35-959 Rzeszow, Poland
[2] Faculty of Chemistry, Rzeszow University of Technology, Al. Powstancow Warszawy 6, 35-959 Rzeszow, Poland
* Correspondence: jml@prz.edu.pl

Table S1. Interpretation of MALDI-TOF spectrum of polyol (CS+GL)+EC.

Entry	Signal position m/z	Relative intensity of signal [%]	Molecular ion structure	Calc. molecular weight [g/mol]
1	96.919	100.00	GL+Na^+	97.027
2	101.035	8.26	GL+OE-H_2O+H^+	101.060
3	112.950	40.31	GL+K^+	113.000
4	114.858	34.78	GLYC+Na^+	115.037
5	120.936	14.86	GL+OE+H^+	119.071
6	140.918	11.29	GL+OE+Na^+	141.053
7	156.923	31.38	GL+OE+K^+	157.027
8	197.006	78.74	GLYC+GL+CH_3OH	198.110
9	212.810	6.84	GLYC+GL+OE+H^+	211.118
10	236.938	5.69	GLYC+3OE-H_2O+ CH_3OH	238.142
11	263.080	6.38	GLYC+2GL+Na^+	263.111
12	293.085	97.34	GLYC+GL+2OE+K^+	293.100
13	307.099	35.42	GLYC+2GL+OE+Na^+	307.137
14	321.115	17.96	4GL+EO-H_2O	322.163
15	351.158	22.93	GLYC+2GL+2OE+H^+	351.163
16	365.134	24.00	4GL+2OE+H_2O	366.189
17	393.937	89.83	2GL+6OE-H_2O	394.220

Description of abbreviations used in the table:

GL – glycidol, GLYC - glycerol OE – oxyethylene group from EC, H_2O – water, K+ – potassium ion from the catalyst K_2CO_3, Na+ – sodium ion, CH_3OH – methanol

Table S2. Interpretation of MALDI-TOF spectrum of polyol (CS+GL+EC).

Entry	Signal position m/z	Relative intensity of signal [%]	Molecular ion structure	Calc. molecular weight [g/mol]
1	96.942	18.71	$GL+Na^+$	97.027
2	101.085	19.86	$GL+OE-H_2O+H^+$	101.060
3	112.861	29.61	$GL+K^+$	113.000
4	114.858	27.80	$GLYC+Na^+$	115.037
5	156.860	17.39	$GL+OE+ K^+$	157.027
6	263.100	5.17	$GLYC+2GL+ Na^+$	263.111
7	291.186	100.00	$GLYC+GL+2OE+K^+$	293.100
8	307.160	39.28	$GLYC+2GL+OE+Na^+$	307.137
9	337.125	23.62	$3GL+3EO- H_2O+ H^+$	337,186
10	351.139	80.22	$GLYC+2GL+2OE+Na^+$	351.163
11	365.170	25.95	$4GL+2OE+H_2O$	366.189
12	381.147	31.53	$3GL+4EO- H_2O+ H^+$	381,213
13	393.950	81.77	$2GL+6OE-H_2O$	394.220

Description of abbreviations used in the table:

GL – glycidol, GLYC - glycerol OE – oxyethylene group from EC, H_2O – water, K+ – potassium ion from the catalyst K_2CO_3, Na+ – sodium ion, CH_3OH – methanol

Table S3. Thermal analysis of foam determined by the dynamic method.

Foam obtained from	Water [%]	$T_{5\%}$[°C]	$T_{10\%}$[°C]	$T_{25\%}$[°C]	$T_{50\%}$[°C]	Tg [°C]
(CS_o+GLYC+GL)+EC	2	221	261	311	382	72
	3	247	266	317	385	73
(CS_O+GL)+EC	2	75	156	186	226	104
	3	75	172	208	284	113
(CS_O+GL+EC)	2	186	222	280	350	100
	3	87	203	263	328	100

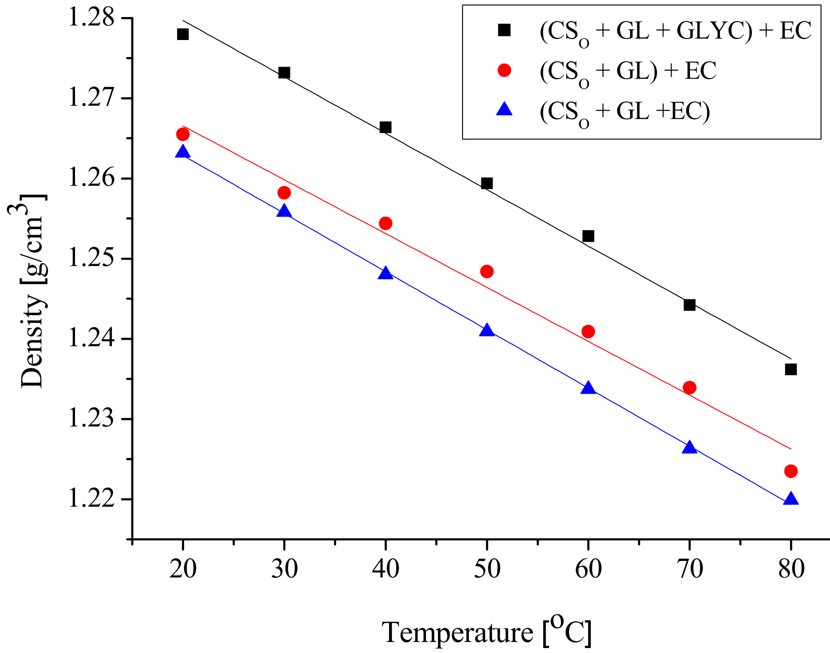

(CS$_O$ + GL + GLYC) + EC
(CS$_O$ + GL) + EC
(CS$_O$ + GL +EC)
Density [g/cm^3]
1.28
1.27
1.26
1.25
1.24
1.23
1.22
20
30
40
50
60
70
80
Temperature [^{O}C]

(a)

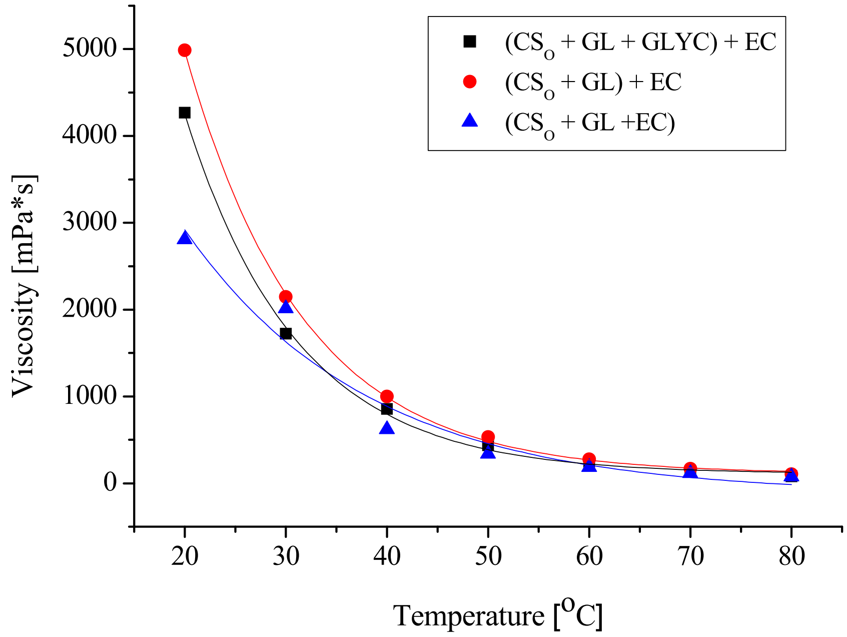

(CS$_O$ + GL + GLYC) + EC
(CS$_O$ + GL) + EC
(CS$_O$ + GL +EC)
Viscosity [mPa*s]
5000
4000
3000
2000
1000
0
20
30
40
50
60
70
80
Temperature [^{O}C]

(b)

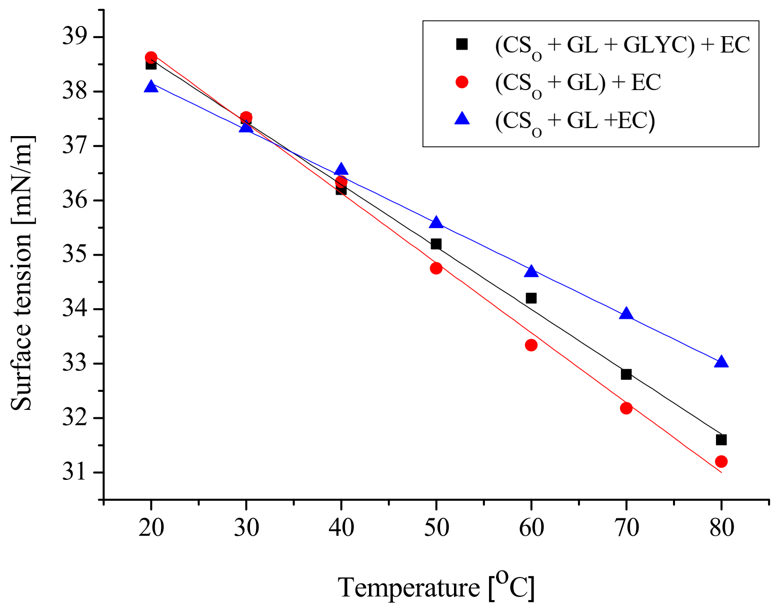

(c)

Figure S1. Changes of density (a), viscosity (b), and surface tension (c) of polyol as a function of temperature.

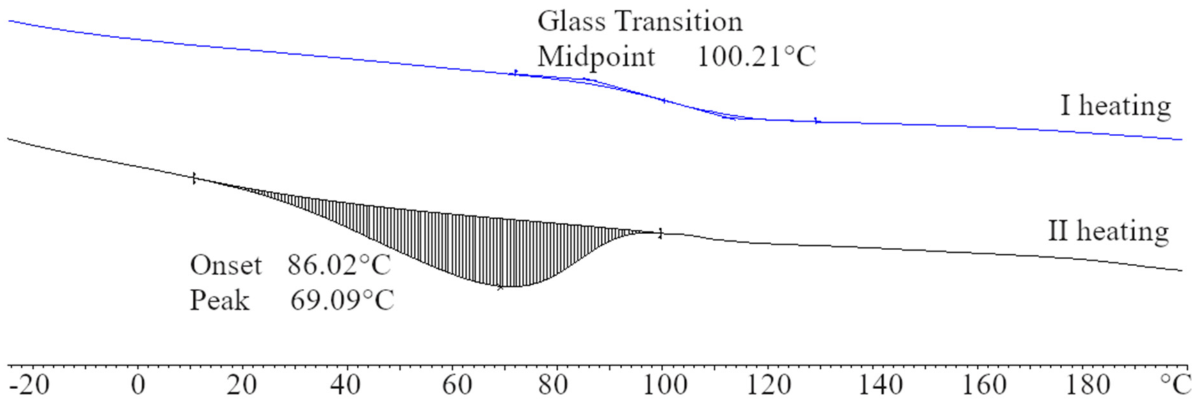

Figure S2. Thermogram of foam obtained from polyol (CS+GL+EC) (2% H_2O/100 g of polyol.
